# Antifungal Activity of Microbial Secondary Metabolites

**DOI:** 10.1371/journal.pone.0025321

**Published:** 2011-09-22

**Authors:** Jeffrey J. Coleman, Suman Ghosh, Ikechukwu Okoli, Eleftherios Mylonakis

**Affiliations:** Harvard Medical School, Division of Infectious Diseases, Massachusetts General Hospital, Boston, Massachusetts, United States of America; New York State Health Department and University at Albany, United States of America

## Abstract

Secondary metabolites are well known for their ability to impede other microorganisms. Reanalysis of a screen of natural products using the *Caenorhabditis elegans*-*Candida albicans* infection model identified twelve microbial secondary metabolites capable of conferring an increase in survival to infected nematodes. In this screen, the two compound treatments conferring the highest survival rates were members of the epipolythiodioxopiperazine (ETP) family of fungal secondary metabolites, acetylgliotoxin and a derivative of hyalodendrin. The abundance of fungal secondary metabolites indentified in this screen prompted further studies investigating the interaction between opportunistic pathogenic fungi and *Aspergillus fumigatus*, because of the ability of the fungus to produce a plethora of secondary metabolites, including the well studied ETP gliotoxin. We found that cell-free supernatant of *A. fumigatus* was able to inhibit the growth of *Candida albicans* through the production of a secreted product. Comparative studies between a wild-type and an *A. fumigatus ΔgliP* strain unable to synthesize gliotoxin demonstrate that this secondary metabolite is the major factor responsible for the inhibition. Although toxic to organisms, gliotoxin conferred an increase in survival to *C. albicans*-infected *C. elegans* in a dose dependent manner. As *A. fumigatus* produces gliotoxin *in vivo*, we propose that in addition to being a virulence factor, gliotoxin may also provide an advantage to *A. fumigatus* when infecting a host that harbors other opportunistic fungi.

## Introduction

Microbial secondary metabolites have provided numerous pharmaceutical agents ranging from antibiotics to immunosuppressive compounds. Synthesis of these low molecular weight compounds is not required for normal growth of the microbe, however these compounds may provide several benefits to the organism. Fungi have the ability to produce a plethora of secondary metabolites, typically dependent on the stage of development of the fungus and environmental factors ranging from nutrient concentrations to light and temperature [1], [2]. Fungi belonging to the genus *Aspergillus* are especially capable of producing a diverse array of these compounds [3], [4]. The filamentous fungus *Aspergillus fumigatus* secretes more than 226 secondary metabolites including commonly studied polyketides, such as cyclic peptides, alkaloids, and sesquiterpenoids [4]. Members of another class of secondary metabolites produced by *A. fumigatus*, termed the epipolythiodioxopiperazines (ETPs), are characterized by an internal disulphide bridge across a diketopiperazine ring, where the first and best characterized member being gliotoxin [5].

*A. fumigatus* spores are ubiquitous in the environment and are commonly inhaled. Invasive aspergillosis usually only effects immune-compromised patients (those with leukemia, transplantation) or patients with other medical conditions such as cystic fibrosis, chronic obstructive pulmonary disease, or severe asthma, as the primary route to an established infection is through the lungs [6]. Among different *Aspergillus* species, only those associated with aspergillosis, such as *A. fumigatus*, *A. terreus*, *A. flavus*, and *A. niger*, produce gliotoxin [7], [8]. Conversely, *A. nidulans*, a saprobe not normally associated with invasive aspergillosis, does not have the secondary metabolite gene cluster necessary to produce gliotoxin or any other ETP [9]. The role of gliotoxin in mammalian virulence is not fully known as conflicting results exist (recently reviewed in [10]). In *A. fumigatus*, the gliotoxin secondary metabolite gene cluster is composed of 12 genes approximately 28 kb in length (the *gli* cluster) [11]. Dioxopiperazine synthase (*GliP*) is required in the first step for the biosynthesis of gliotoxin generating the characteristic diketopiperizine ring [5], [12].

Factors which enable *A. fumigatus* to colonize and remain established within the host by competing for limited available nutritional resources are currently unknown; however gliotoxin has potent antifungal activity against *Candida albicans*, *Cryptococcus neoformans*, and other fungi [13]. This is interesting because pathogenic fungi, such as *C. albicans* and *C. neoformans*, primarily infect or colonize hospitalized patients and particularly the same patient population as *A. fumigatus*, providing an environment conducive of pathogen-pathogen interactions between these fungi, in particular within the pulmonary system. For example, concurrent co-infection/colonization of *Aspergillus* spp. and *Candida* spp. can occur in patients [14]. Moreover, *Candida* spp. can colonize the respiratory tract of hospitalized patients, and the ability of a fungus such as *A. fumigatus* to compete against a previously established *Candida* spp. colonization may be necessary for the second pathogen to develop an infection.

Here we reanalyzed the results from a recently published *in vivo Candida*-infected nematode survival assay to identify secondary metabolites capable of prolonging nematode survival. We found that two members of the ETP class of secondary metabolites we able to significantly increase nematode survival after infection with *C. albicans*. As pathogenic fungi are capable of producing these compounds within a host, the inhibitory action of gliotoxin against *C. albicans* was further studied. This research investigates the potential antagonistic activities mediated by secondary metabolites that may be occurring among fungi within a host.

## Results and Discussion

### Secondary metabolites promoting *Candida*-infected *Caenorhabditis elegans* survival

**Figure 1:**
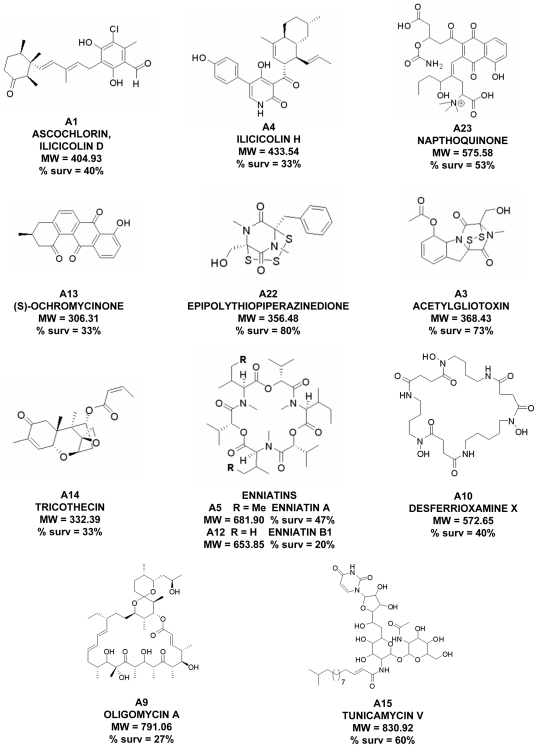
Compound structures of secondary metabolites able to confer an increase in survival to *Candida*-infected nematodes. The maximum nematode survival (%) and molecular weights are indicated for each of the compounds. Structures were provided by Analyticon Discovery.

The *C. albicans*-*C. elegans* antifungal discovery assay allows simultaneous assessment of the ability of a compound to promote survival of infected nematodes and indicate if there is any associated potential toxicity [15]. Previously, a high-throughput screen of 2,560 natural products from the Analyiticon Discovery compound collection (www.ac-discovery.com) was conducted that identified several plant produced saponins that confer an increase in *Candida*-infected nematode survival [16]. In addition to these saponins, reanalysis of this screen also identified twelve microbial secondary metabolites that were able to prolong nematode survival and may have antifungal activity (Figure 1; Table 1). These compounds were produced by bacteria and fungi, and several natural products that were closely related to known antifungal compounds were identified in this screen. Compounds conferring *C. albicans*-infected nematode survival rates greater then 40% were chosen for further discussion.

**Table 1:** Compounds identified in a screen of natural products containing secondary metabolites.

Compounds	Percent nematode survival	Mode of action	Reference(s)
Epipolythiopiperazinedione (A22)	80%	Binding to free protein thiols and generation of ROS	[5]
Acetylgliotoxin (A3)	73%	Binding to free protein thiols and generation of ROS	[5]
Tunicamycin V (A15)	60%	Inhibits the transfer of *N*-acetylglucosamine to a lipid intermediate	[18], [20]
Napthoquinone (A23)	53%	Complex naphthoquinine; diverse biological properties	
Enniatin A (A5)	47%	Disrupt cellular physiological cation concentrations	[22], [47]
Desferrioxamine X (A10)	40%	Hydroxamate siderophore – specific chelators of iron(III)	[23], [24]
Ascochlorin/Ilicicolin D (A1)	40%	Inhibitor of the cytochrome *bc*1 complex in mitochondria	[27], [28]
Ilicicolin H (A4)	33%	Inhibitor of the cytochrome *bc*1 complex in mitochondria	[29], [48]
(S)-Ochromycinone (A13)	33%	Multiple biological properties, but exact MOA is unknown	[49]
Tricothecin (A14)	33%	Inhibits translation by binding to ribosomes	[50], [51]
Oligomycin A (A9)	27%	Inhibitor of F_0_ domain of the H^+^-ATP synthase	[52]
Enniatin B1 (A12)	20%	Disrupt cellular physiological cation concentrations	[22], [47]

**Figure 2:**
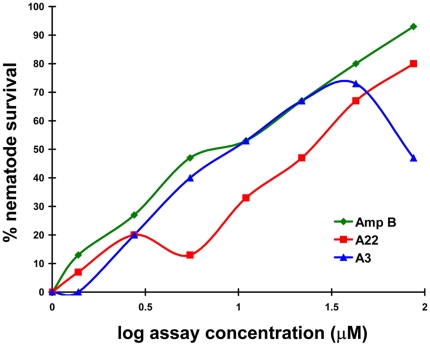
Dose response of two ETP compounds from the *C. albicans*-*C. elegans* antifungal discovery assay. A22 and A3 were as effective as amphotericin B in increasing nematode survival, however the decrease observed for compound A3 suggests there maybe toxicity associated with the compound at higher concentrations. The dose response experiment was conducted a single time as previously reported in Okoli et al., 2009.

Interestingly, two members (A3 and A22) of the ETP family of secondary metabolites provided the highest worm survival of the natural products screened (Figure 1; Table 1) and A22 (80%) and A3 (73%) promoted greater than 65% nematode survival (Figure 1). Of note is that the dose response for nematode survival for both of these ETP compounds was comparable to amphotericin B (Figure 2). Toxicity for acetylgliotoxin was observed in the *C. elegans* assay at higher concentrations (>16 µg/ml; Figure 2), no toxicity was seen at the highest concentration tested for A22 (31 µg/ml; Figure 2). A number of ETP secondary metabolites are synthesized by fungi [5]. The most common form of this class of compounds contains a disulphide bridge, but sometimes versions containing one, three, or four sulfur atoms are also produced [5]. The disulphide bridge containing form of A22 has been previously isolated from *Hyalodendron* sp. and identified as hyalodendrin [17], one of the few ETP compounds produced by a basidiomycete [5].

Among the other secondary metabolites, compound A15 (tunicamycin V) is a member of the tunicamycin family of antimicrobial agents with a modified fatty acid side-chain (Figure 1) [18]. Tunicamycin is a mixture of homologous nucleoside compounds and inhibits N-linked glycosylation [19]. Tunicamycin is highly toxic if swallowed, and targets the liver and nerves. However, the (*E*)-13-methyltetradec-2-enoic acid substituted analog tunicamycin V gave up to 60% protection to the worms and showed no signs of toxicity in the screen. Several tunicamycin homologues have previously been reported to inhibit *C. albicans*, and their degree of toxicity varied depending on the fatty-acid side chain [20].

Importantly, distinct structure activity relationships were discernible between several closely related analogs in the antifungal screen. For example, two members of the enniatin family of natural products, enniatin A (A5) and enniatin B1 (A12), produced by *Fusarium* spp. were active in the assay [21]. This class is characterized by the alternating arrangement of ester and amide linkages comprising an 18 membered macrocycle. Enniatin A and enniatin B1 differ by only two methyl substitutions and demonstrated protective activity with the isovaleroyl substituted enniatin A being twice as effective, 47% vs 20% respectively (Figure 1). The protective activity of the enniatins is reflected in their MIC, as enniatin A inhibits *C. albicans* growth at half the concentration as enniatin B1 [22]. These compounds did not display toxicity to the worms in subsequent dose-response experiments, however the enniatins provided a low degree of protection to the worms.

Another notable secondary metabolite, desferrioxamine X belongs to the well-studied class of hydroxamate siderophores [23], [24]. This class of cyclic hexadentate siderpohores are specific chelators of iron(III) and are produced by a variety of bacteria and fungi under iron deficient conditions. These hydroxamate based macrocycles sequester and solubilize iron(III) which is then actively transported into the organism. While siderophores have been utilized in humans for iron and aluminum overload therapy and some antibiotic applications, use of desferrioxamine in *C. albicans* treatment has not been extensively studied. However, desferrioxamine increases severity of mycoses caused by some fungi, in particular pathogenic members of the order Mucorales, as the fungi are able to uptake and utilize the iron chelated by the siderophore, although there appears to be no significant difference with *C. albicans* [25], [26]. While the overall protective effect was moderate, 40%, desferrioxamine X showed no toxicity to the worms at higher concentrations.

Finally, the secondary metabolite ascochlorin (A1), also referred to as ilicicolin D, is an prenyl-phenol compound that was originally identified in extracts of the fungus *Ascochyta viciae* [27]. Ascochlorin inhibits mitochondrial electron transport via binding to the Q_i_ and Q_o_ sites of the cytochrome *bc*_1_ complex [28]. Interestingly, another secondary metabolite identified in the screen, ilicicolin H (A4) produced by the fungus *Cylindrocladium iliciola*, also acts by inhibiting the cytochrome *bc*_1_ complex, however this molecule binds at the Q_n_ site [29]. Both ascochlorin and ilicicolin H conferred a similar *C. albicans*-infected nematode survival rate (40% and 33%, respectively; Figure 1 and Table 1).

### Secondary metabolites from *A. fumigatus* inhibit other opportunistic fungi

The secondary metabolites produced by *A. fumigatus* were chosen for additional investigation. This fungus was chosen for further studies with *C. albicans* and *C. neoformans* because of the numerous secondary metabolites known to be synthesized and have been characterized, including gliotoxin, the deacetylated version of A3. Although rare, concurrent fungal infection/colonization between *A. fumigatus* and *C. albicans* or *C. neoformans* have been documented [14]. Isolation of *Candida* spp. from respiratory specimens is generally not indicative of colonization, but rather is the result of contamination of the bronchoscope from the gastrointestinal tract during the examination procedure. However, it is notable that in a study of postmortem examinations, six (4.8%) revealed concurrent *Aspergillus* spp. and *Candida* spp. infection and one with *Aspergillus* spp. and *C. neoformans* (<1%) [14].

**Figure 3:**
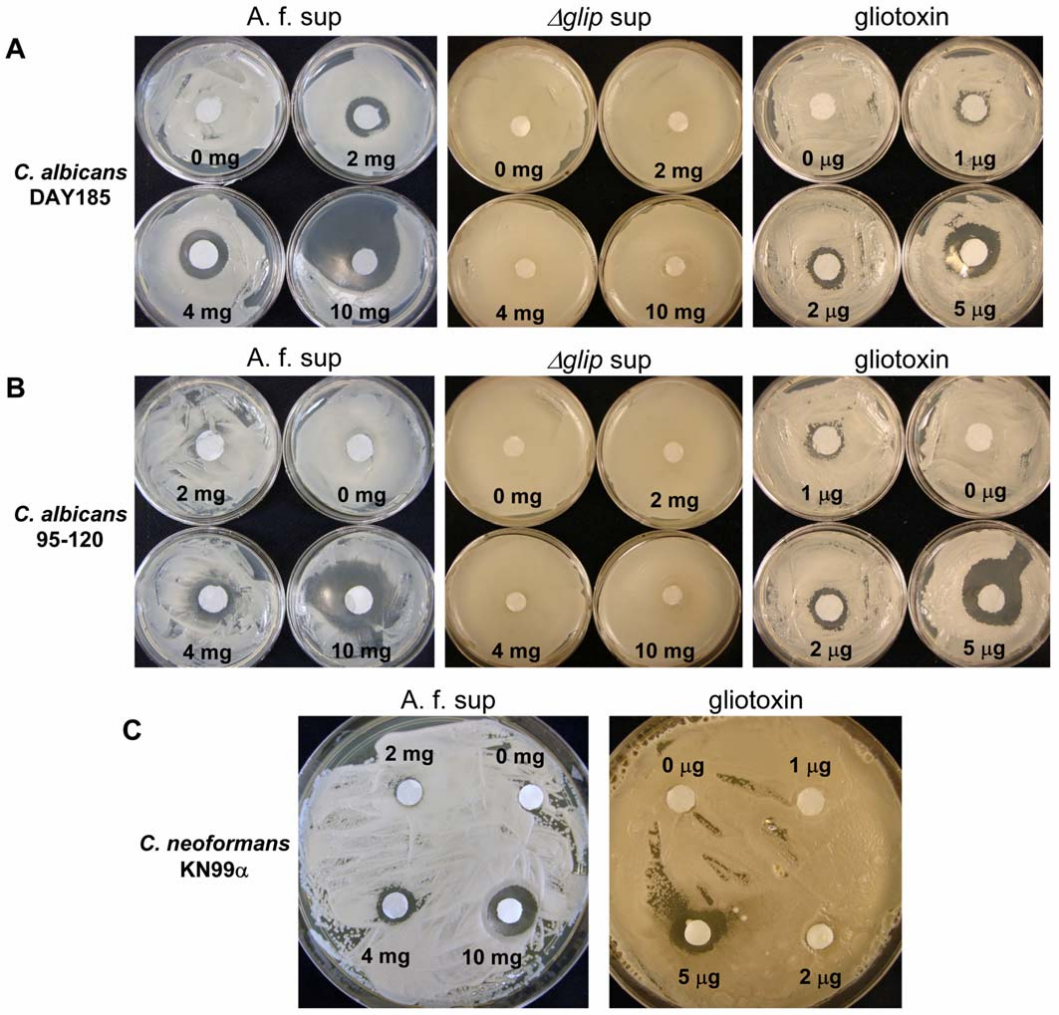
Inhibition of *C. albicans* by A.f. supernatents and gliotoxin. *C. albicans* strains DAY185 (**A**) and 95–120 (**B**) grown on Spider medium at 37°C overnight in the presence of discs containing the indicated amounts of AF293 supernatent (A.f. sup); supernatant from the ΔgliP mutant unable to synthesize gliotoxin (g*liP* sup); or pure gliotoxin. The growth of *C. albicans* strain 98–145 in the presence of the treatments was similar to 95–120 (data not shown). **C.** *C. neoformans* wild type strain KN99α grown on YPD at 30°C overnight in the presence of discs containing indicated amounts of A.f. sup or gliotoxin.

When *A. fumigatus* was co-inoculated with *C. albicans* or *C. neoformans* on plates containing Spider medium at 37°C, yeast colonies proximal to *A. fumigatus* were unable to grow (data not shown), suggesting that *A. fumigatus* produces a secreted toxic agent to both *C. albicans* and *C. neoformans* at the stationary phase. In order to identify if any secondary metabolites of *A. fumigatus* were responsible for the growth inhibition to *C. albicans* and *C. neoformans*, *A. fumigatus* supernatant (AFS) was collected. As little as 2 mg of AFS was able to form a zone of inhibition around *C. albicans* strains DAY185, 95–120, or 98–145 on plates containing Spider medium and grown at 37°C (Figure 3); a zone of inhibition was also observed with *C. neoformans* KN99α on YPD plates grown at 30°C (Figure 3). AFS was also able to produce a slight zone of inhibition around the *C. albicans* strains at 30°C on YPD plates (data not shown).

To further assess the nature of the secreted product(s) in the supernatant that are responsible for the inhibition of *C. albicans* and *C. neoformans*, the supernatant was heated at 60°C for 2 hours to inactivate potential enzymatic activity. The inhibitory activity of the heat inactivated supernatant (HI-AFS) was assayed on *C. albicans* DAY185 and was able to produce a zone of inhibition on plates containing Spider medium and grown at 37°C (Figure S1), similar to the results observed with AFS. As a negative control the fungal inhibitory potential of the *C. neoformans* supernatant was assessed on *C. albicans* growth. The *C. neoformans* supernatant was unable to produce a zone of inhibition with *C. albicans* DAY185 when grown on either YPD at 30°C or Spider medium at 37°C (Figure S2). Taken together, these data suggest that AFS contains heat stable product(s), possibly secondary metabolites, which were secreted from the fungus capable of inhibiting the growth of *C. albicans* and *C. neoformans*.

### Gliotoxin is the major secondary metabolite of *A. fumigatus* that is toxic to *C. albicans* and *C. neoformans*

The *A. fumigatus ΔgliP3* mutant strain that does not produce gliotoxin [12] was used to investigate if gliotoxin produced by *A. fumigatus* is responsible for the inhibitory activity of the supernatant to *C. albicans* and *C. neoformans*. Unlike the supernatant from the wild type AF293 strain, the supernatant of the *ΔgliP* isolate failed to produce a zone of inhibition for either *C. albicans* strains DAY185, 95–120, 98–145, or *C. neoformans* KN99α, demonstrating that gliotoxin was responsible for the zone of inhibition observed with all the *C. albicans* and *C. neoformans* strains. Further studies on the inhibitory activity of commercially available gliotoxin supports that this compound was responsible for the zone of inhibition observed with *C. albicans* and *C. neoformans*. The area of the zone of inhibition produced by pure gliotoxin was in a dose dependent manner as observed with AFS (Figure 3). Pure gliotoxin produced a clear zone of inhibition of *C. albicans* on Spider medium grown at 37°C as observed previously with AFS, but the small zone of inhibition was not apparent with *C. albicans* on YPD media grown at 30°C suggesting gliotoxin may not be responsible for the slight observed inhibition in this condition (data not shown). These studies demonstrate that gliotoxin is the major component involved in the inhibitory activity of the *A. fumigatus* supernatant when grown in Spider medium at 37°C.

### The effects of gliotoxin in *C. albicans* and *C. neoformans*

Pure gliotoxin and AFS were used to find the minimum inhibitory concentration (MIC) *in vitro* of *C. albicans* strains DAY185, 95–120, 98–145, and *C. neoformans* strain KN99α. The MIC of gliotoxin was 2.0 µg/ml for *C. albicans* and 4.0 µg/ml for *C. neoformans* (Table 2), whereas the MIC of AFS was 3.2 mg/ml for all the strains tested (Table 2). In murine studies, gliotoxin was able to be accumulated in lung tissue to a mean concentration of ∼4 µg/g and was also detected in the sera of the animals, although at a significantly reduced concentration, 36 ng/mL [30]. Gliotoxin was capable of being detected in several patient serum samples where the concentration of one sample was 785 ng/mL (range 166–785 ng/mL) [30], suggesting gliotoxin can accumulate in patients with invasive aspergillosis at a concentration capable of inhibiting other fungal pathogens.

**Table 2:** MIC and EC50 of gliotoxin and A. fumigatus supernatant as assessed in the C. elegans-C. albicans assay.*

	MIC *in vitro*				EC_50_
	DAY185	95–120	98–145	KN99α	DAY185
Gliotoxin (µg/ml)	2.0	2.0	2.0	4.0	2.0
*A.fumigatus* supernatant (mg/ml)	3.2	3.2	3.2	3.2	1.0

*The MICs of gliotoxin were conduced in duplicate, while the MICs of the *A. fumigatus* supernatant and the EC_50_ are based on a single measurement.

Gliotoxin is produced by a number of fungi [5], although whether gliotoxin is produced by *C. albicans* is not known, as conflicting studies exist suggesting gliotoxin is produced by some strains of *C. albicans* [31], however subsequent studies have shown that the fungus does not produce the ETP [32]. In support of the lack of gliotoxin production in *C. albicans*, the genome of the fungus does not contain a secondary metabolite gene cluster predicted to synthesize an ETP [5], and this study demonstrates that *C. albicans* is highly susceptible to gliotoxin (Figure 3; Table 2).

The toxicity of gliotoxin is possibly due to several mechanisms. Gliotoxin has the potential to induce production of reactive oxygen species (ROS) by a intracellular redox cycle, where the reduced compound oxidizes to reform the disulfide bridge, producing hydrogen peroxide and superoxide in the process [5]. ETP compounds also have the potential to react with numerous cellular proteins which have exposed cystine residues, and therefore have no “specific” mode of action [5]. In addition, it has been demonstrated that in mammalian cells the reduced form of gliotoxin is unable to cross the plasma membrane, resulting in accumulation inside the cell, and therefore the intracellular concentration of gliotoxin is several orders of magnitude higher and predominantly in the reduced form [33]. The hydrogen peroxide produced by the redox cycle of gliotoxin has been implicated in causing single- and double-stranded DNA breaks [34], and therefore some of the antifungal activity of gliotoxin may potentially also be derived by the damage of fungal DNA.

**Figure 4:**
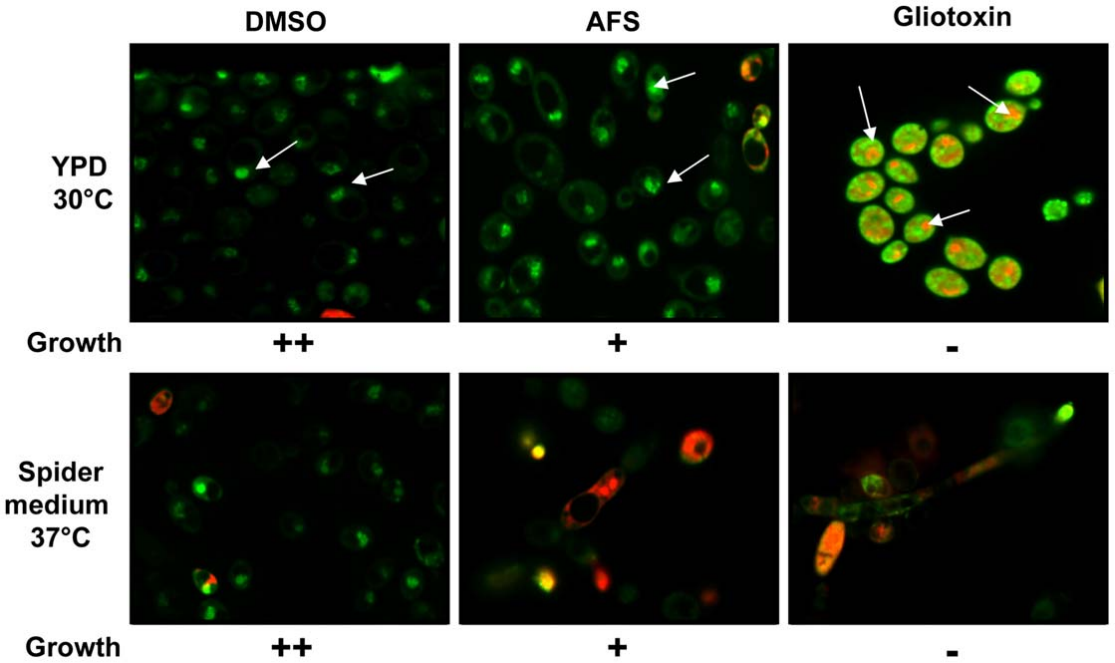
Assessment of *C. albicans* viability after treatment with gliotoxin. Confocal laser microscopy of *C. albicans* after staining with the Live/Dead staining system, whereby dead cells stain red and live cells stain green. *C. albicans* strain DAY185 was grown overnight in YPD at 30°C or in Spider medium at 37°C, treated with DMSO, *A. fumigatus* supernatent (AFS; 3.2 mg/ml), or gliotoxin (2.0 µg/ml). The yellowish color is reflective of co-localization of both red and green dyes suggesting the cells have increased permeability and maybe potentially dying or already dead. White arrows showing intact nucleus with green color in DMSO and AFS treated cells, and partially dead nucleus with yellowish color in gliotoxin treated cells. There was growth (++) in case of DMSO treated cells, partial growth (+) in A.f. sup treated cells, and no growth (-) in gliotoxin treated cells. Gliotoxin treated cells were centrifuged before microscopy.

The effects of AFS and gliotoxin on *C. albicans* DAY185 were further evaluated using the BacLight live-dead staining kit (Molecular probes). Using this system, live fungi with intact membranes fluoresce green, while dead fungi with damaged membranes fluoresce red. The live-dead staining reflects the previous observation where DMSO treated cells were alive (green), and AFS (3.2 mg/ml) and gliotoxin (2.0 µg/ml) treated cells were dead (red) in Spider medium at 37°C. At 30°C, *C. albicans* yeast cells treated with DMSO and AFS (3.2 mg/ml) were alive (green, Figure 3). In contrast, gliotoxin treated cells did not grow and the nuclear contents were yellowish with the surrounding cytosol green (Figure 4, gliotoxin treated cells in YPD at 30°C).

The inhibitory activity of gliotoxin (and AFS) treated *C. albicans* cells was consistently higher against cells grown in Spider medium compared to cells grown in YPD medium (Figures 3, 4 and S1). Although the reason(s) for the observed differences are unknown, we speculate that gliotoxin may have increased activity against cells with a filamentous morphology which is favored by growing *C. albicans* in Spider medium. Additionally, physiological properties such as permeability or increased ROS generation could potentially be contributing to the antifungal activity of gliotoxin when grown under this condition.

*A. fumigatus* is resistant to the effects of gliotoxin, as one of the genes in the *gli* cluster, *gliT*, encodes a reductase that confers a high level of self-protection to the compound [35]. Additionally, another member of the gene cluster, *gliA*, encodes a major facilitator transporter that has been shown to be involved in efflux of the ETP [36]. Therefore, at least two mechanisms exist in *A. fumigatus* that confer tolerance to gliotoxin that are absent in most other fungi, including *C. albicans* and *C. neoformans*. In general there are fewer efflux transporters in *C. albicans* and *C. neoformans* when compared to *A. fumigatus* [37], and therefore are less likely to have the capability to transport gliotoxin out of the cell. Of note is that two fluconazole resistant isolates of *C. albicans* were as susceptible to gliotoxin as the wild-type isolate suggesting the efflux pumps conferring resistance to fluconazole do not confer resistance to gliotoxin (Figure 3; Table 2). The lack of mechanisms able to confer resistance to gliotoxin may account for the high level of inhibitory activity of the compound.

### *C. elegans* survival as a marker for the evaluation of the antifungal activity of gliotoxin

**Figure 5:**
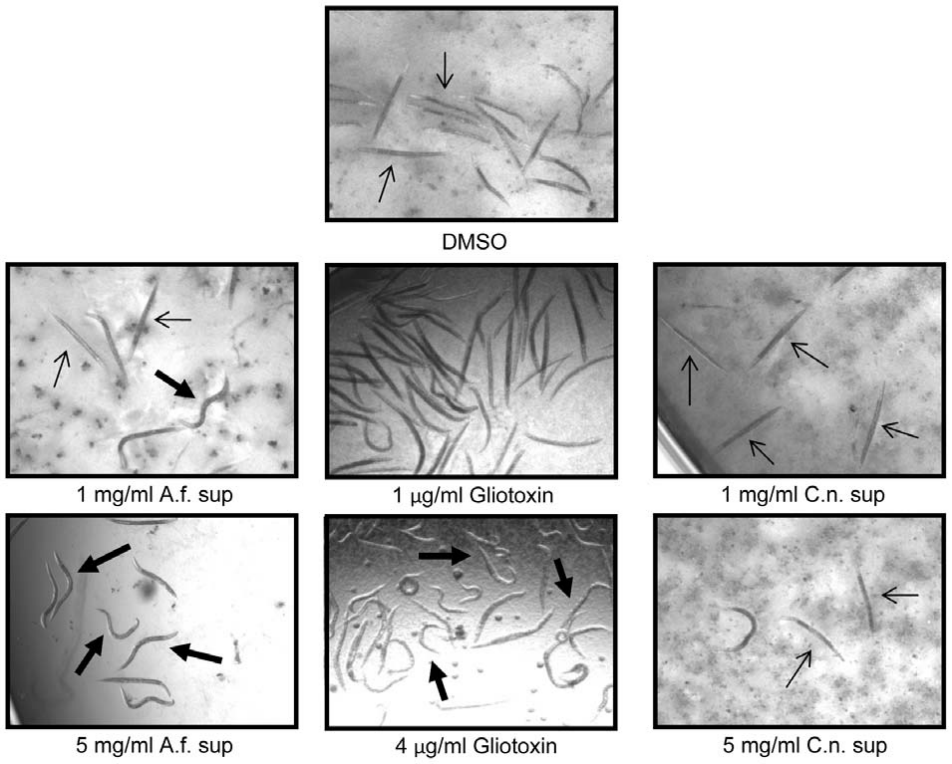
*C. elegans*-*C. albicans* co-infection assay to assess the ability of gliotoxin to promote nematode survival. Representative assay wells from the *C. elegans*-*C. albicans* infection assay. The wells were treated with DMSO (negative control) or the indicated concentrations of A.f. supernatant (A.f. sup), gliotoxin, or *C. neoformans* supernatant (C.n. sup). Sinusoidal shaped worms are alive (thick black arrows) and rod shaped nematodes are dead (thin arrows).

The *C. elegans*-*C. albicans* model system was used to evaluate the efficacy of AFS and gliotoxin using nematode survival as a method to gauge the antifungal activity of the compound. Although other invertebrate host models exist [38], [39] *C. elegans* is an ideal heterologous host to evaluate the effects of gliotoxin, as the nematode lacks a NF-κB homolog while other immune response pathways remain intact [40], [41], and therefore some of the immunosuppressive activity derived by inactivation of this transcription factor does not interfere with the evaluation of the antifungal activity of gliotoxin. AFS and gliotoxin were able to inhibit the growth of *C. albicans*, prolonging the survival of *C. elegans* (Figure 5). The effective concentration which resulted in 50% survival of nematodes (EC_50_) of pure gliotoxin and AFS were determined. The EC_50_ of AFS and gliotoxin were 1.0 mg/ml and 2.0 µg/ml, respectively (Table 2 and Figure 5). The highest concentration of AFS used in the *C. elegans* assay was 12.8 mg/ml (Table 2) a concentration that appeared to be non-toxic to the nematode. As a control, the supernatant of *C. neoformans* was unable to inhibit the growth of *C. albicans* and prolong the survival of *Candida*-infected *C. elegans* (Figure 5).

### Concluding remarks

Relatively few studies exist describing the interactions between fungi, and usually these studies pertain to use of a fungus to control a phytopathogenic fungus in agriculture. As the opportunistic fungal pathogens *C. albicans* and *A. fumigatus* may exist within the same infected individual or share a similar environmental niche, it is imperative to understand the fungal-fungal interactions that maybe taking place. Additionally, the inhibitory activity of gliotoxin against pathogenic bacteria suggests this compound may also have a role in the interaction between medically-relevant bacteria and fungi capable of synthesizing the compound, as these interactions may occur more frequently [42], [43]. These interactions create a competition between these microbes in order to obtain limited resources, and secondary metabolites provide a competitive advantage to the microbe harboring them. While gliotoxin production is related to virulence within a susceptible host, this study indicates it may also facilitate *A. fumigatus* colonization and maintenance within an individual by inhibition of other fungal pathogens that may exist within the host.

## Materials and Methods

### Media and strains

*C. albicans*, *C. neoformans*, and *A. fumigatus* strains used in the assays are listed in Table S1. Yeast strains were grown on yeast extract-peptone-dextrose (YPD) (Difco) plates or in YPD liquid media containing kanamycin (45 µg/ml), ampicillin (100 µg/ml), and streptomycin (100 µg/ml) at 30°C. The inoculated liquid media were grown over night on a rotary shaker at 225 rpm. The cells were centrifuged, washed three times with phosphate buffered saline (PBS) and re-suspended in PBS at the required concentrations for experiments. The zone of inhibition assays were conducted on either YPD plates at 30°C or on plates containing Spider medium [44] at 37°C.

### Preparation of culture supernatants

*A. fumigatus* and *C. neoformans* culture supernatants were obtained by growing single isolated colonies in glycerol-arginine-yeast extract media [45] for 6–7 days at 30°C. The liquid supernatant was then centrifuged, filter sterilized, and lyophilized to obtain the crude concentrated fungal supernatants. Dried fungal supernatants were then weighed and dissolved in an appropriate amount of DMSO for use in subsequent assays. Stock solutions of the supernatants were prepared at a concentration of 200 mg/ml.

### Minimal inhibitory concentration assay

The determination of the lowest concentration of the compounds with antifungal activity was accomplished using two-fold serial dilutions of the test compounds in RPMI 1640 media at 35°C for 24 hours [46]. The wells were assessed by a 96-well plate reader (V_max_ kinetic microplate reader, Molecular Devices, Sunnyvale, CA) to determine the concentration exhibiting *in vitro* inhibition of *C. albicans* growth.

### Live-Dead staining

The viability of *C. albicans* in the presence of fungal supernatants and gliotoxin was assessed by using the BacLight LIVE/DEAD staining system according to the manufacturer's protocol (Molecular Probes, Carlsbad, CA). The cells which retained the green fluorescence color were live whereas the red fluorescent cells were considered dead.

### EC_50_ assays

To measure the EC_50_ of gliotoxin and fungal supernatants on worms, the *C. albicans*-*C. elegans* co-inoculation assay was performed using the *C. elegans glp-4*;*sek-1* double mutant in all assays [15]. Nematodes were maintained on nematode growth medium with *Escherichia coli* strain OP50 as the food source. The screen medium was 20% brain heart infusion medium (BHI, Difco) in M9 buffer containing antibiotics kanamycin (90 µg/ml), ampicillin (200 µg/ml), and streptomycin (200 µg/ml). M9 buffer was used to wash the worms as needed and for the diluting the screen media.

At the end of the incubation period, the entire wells were imaged, visually analyzed for *in vitro* fungal growth, followed by visual scoring of live and dead worms based on worm shape, as live worms appear sinusoidal and dead worms are rod shaped. The worms were also tested by using a platinum pick to score live or dead. For the determination of EC_50_, the test compounds were serially diluted two-fold. Half the maximum effective concentration that conferred 50% survival of the worms was determined as the EC_50_.

## Supporting Information

Figure S1 **Inhibition of** ***C. albicans*** **by heat inactivated** ***A.fumigatus*** **supernatant.** *C. albicans* strain DAY185 was grown on YPD media at 30°C and Spider medium at 37°C overnight in the presence of discs containing heat inactivated supernatent from isolate AF293 (HI-AFS) at the indicated concentrations.
(TIF)

Figure S2 **Absence of inhibition of** ***C. albicans*** **growth by** ***C. neoformans*** **supernatent.** *C. albicans* strain DAY185 was grown on YPD media at 30°C and Spider medium at 37°C overnight in the presence of discs containing the supernatant of *C. neoformans* strain KN99α (CNS) and the heat inactivated supernatant of *C. neoformans* (HI-CNS) at the indicated concentrations.
(TIF)

Table S1 **Fungal isolates used in this study.**
(DOC)
