# Supplementary figures and images for: Antifungal Activity of Microbial Secondary Metabolites
Source: PLoS One. 2011 Sep 22;6(9):e25321. doi: 10.1371/journal.pone.0025321 (PMC3178648; doi:10.1371/journal.pone.0025321)

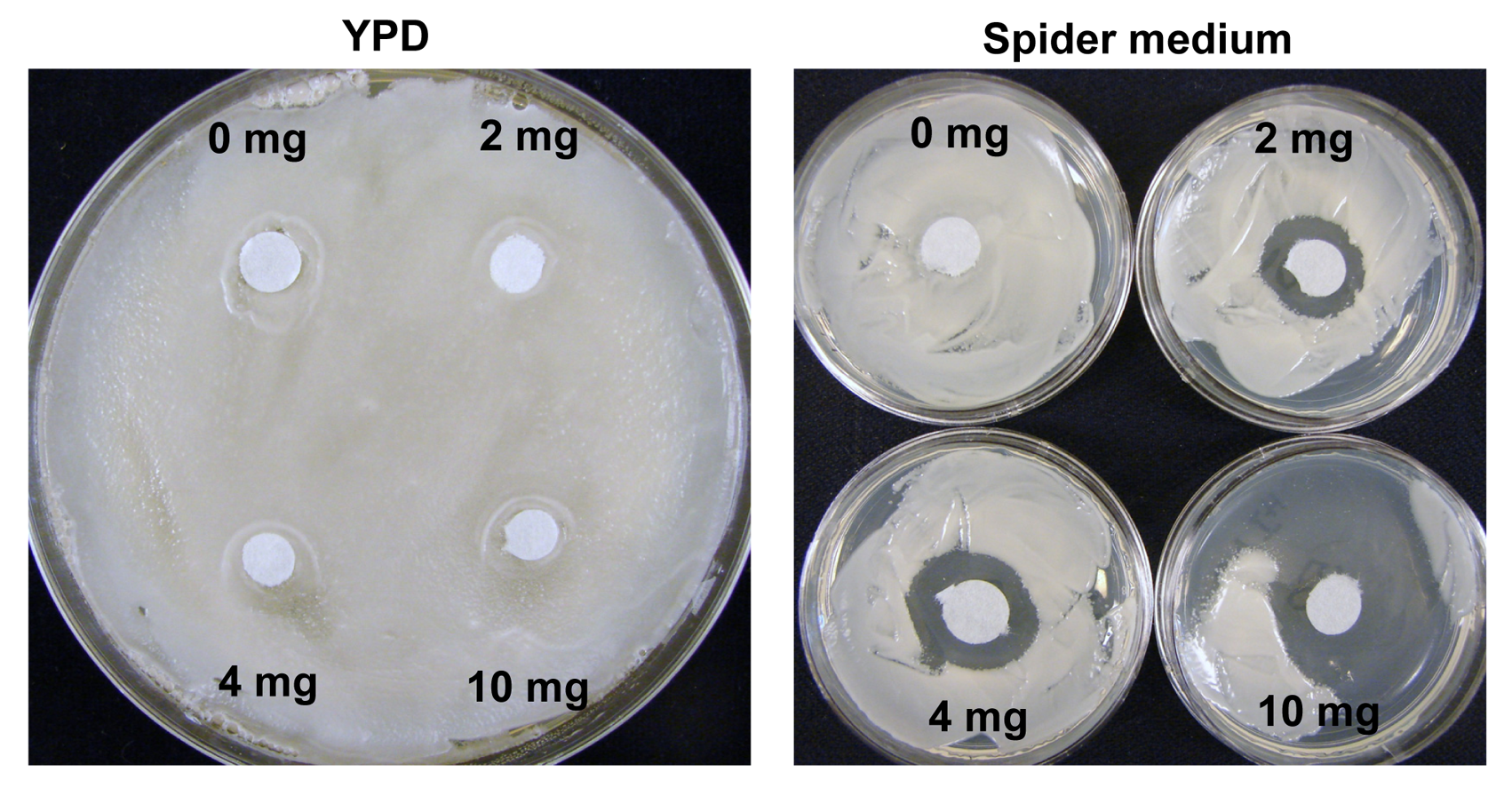

Supplement: Figure S1 — Inhibition of C. albicans by heat inactivated A.fumigatus supernatant. C. albicans strain DAY185 was grown on YPD media at 30°C and Spider medium at 37°C overnight in the presence of discs containing heat inactivated supernatent from isolate AF293 (HI-AFS) at the indicated concentrations. (TIF) [file pone.0025321.s001.tif]

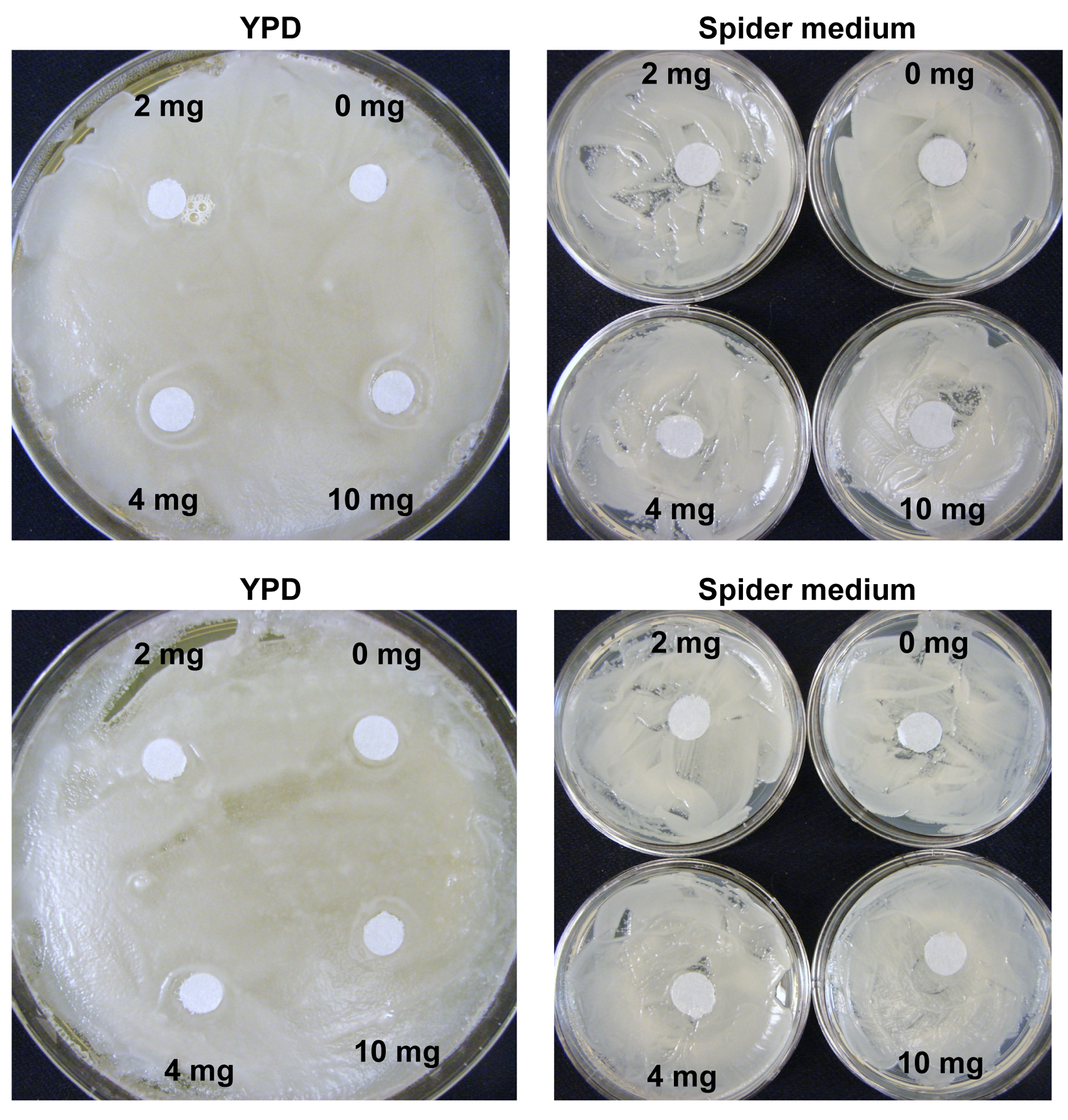

Supplement: Figure S2 — Absence of inhibition of C. albicans growth by C. neoformans supernatent. C. albicans strain DAY185 was grown on YPD media at 30°C and Spider medium at 37°C overnight in the presence of discs containing the supernatant of C. neoformans strain KN99α (CNS) and the heat inactivated supernatant of C. neoformans (HI-CNS) at the indicated concentrations. (TIF) [file pone.0025321.s002.tif]
