# Supplementary material for: Antifungal Activity of Microbial Secondary Metabolites
Source: PLoS One. 2011 Sep 22;6(9):e25321. doi: 10.1371/journal.pone.0025321 (PMC3178648; doi:10.1371/journal.pone.0025321)
Supplement: Table S1 — Fungal isolates used in this study. (DOC) [file pone.0025321.s003.doc]

Table S1.

| **Table S1.** Fungal isolates used in this study. | | |
| --- | --- | --- |
|  | **Characteristics** | **Reference** |
| ***C. albicans*** |  |  |
| DAY185 | Derivative of SC5314, wild-type | [52] |
| 95-120 | Fluconazole resistant clinical isolate | [53] |
| 98-145 | Fluconazole resistant clinical isolate | [53] |
| ***C. neoformans*** |  |  |
| KN99α | Derivative of H99, *MATα* | [54] |
| ***A. fumigatus*** |  |  |
| AF293 | Sequenced wild-type isolate | [55] |
| ΔgliP3 | Mutant unable to synthesize gliotoxin | [12] |
